# A case of SAPHO syndrome with *Staphylococcus saprophyticus* and *Cutibacterium acnes* osteitis

**DOI:** 10.1093/rap/rkz051

**Published:** 2020-02-06

**Authors:** Mara Waters, Sigmund Krajden, Jonathan Stein, Amr Elmaraghy, Ari Eisen, Zared Aziz

**Affiliations:** 1 Department of Medicine, University of Toronto, Toronto, ON, Canada; 2 Department of Medicine, St Joseph’s Health Centre, Toronto, ON, Canada; 3 Department of Surgery, St Joseph’s Health Centre, Toronto, ON, Canada; 4 Division of Orthopaedic Surgery, Department of Surgery, University of Toronto, Toronto, ON, Canada; 5 Department of Diagnostic Imaging, St Joseph’s Health Centre, Toronto, ON, Canada; 6 Department of Laboratory Medicine, St Joseph’s Health Centre, Toronto, ON, Canada

**Key message**

- *Staphylococcus saprophyticus* may be considered as an infectious trigger of SAPHO syndrome.

Sir, SAPHO syndrome is a rare inflammatory cutaneous and osteoarticular condition. *Cutibacterium* (formerly *Propionibacterium*) *acnes* has increasingly been recognized as a pathogen in the development of auto-inflammatory bone disorders [1]. *Staphylococcus saprophyticus* has not previously been reported as a cause of osteitis and hyperostosis in SAPHO syndrome.

We describe the case of a 26-year-old man with a 13-year history of progressive pain in his left clavicle. At 13 years of age, he developed facial acne associated with transient pain in his right hip. Since that time, he experienced episodic and progressive left clavicular pain and swelling. He had no constitutional symptoms. He had previously been investigated several years earlier at a different facility, but had no resolution of his symptoms. He was originally born in Poland and moved to Canada at 5 years of age. Past medical history was unremarkable, and there was no family history of SAPHO syndrome. On examination, there was swelling and tenderness of the medial left clavicle involving the sternoclavicular joint and manubrium, with no other joint involvement. Dermatological examination demonstrated extensive acneiform lesions on the face, chest and back, with no palmoplantar pustulosis.

**Fig. 1:**
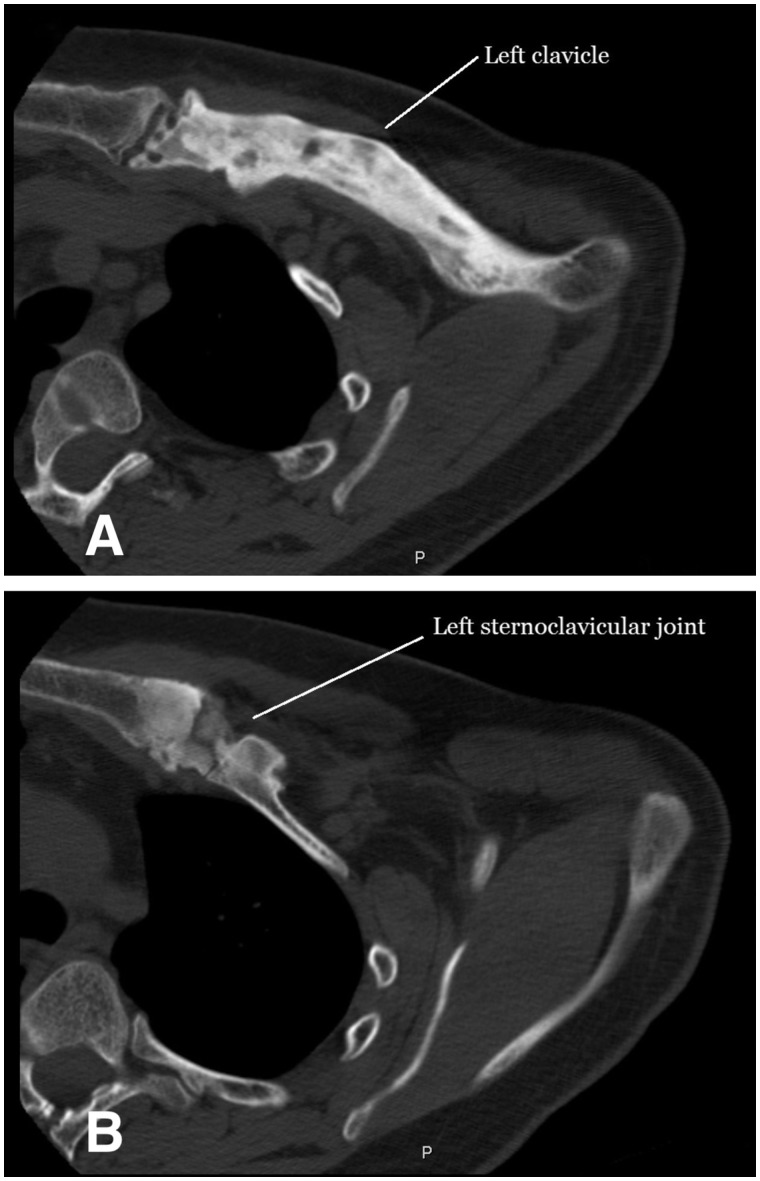
CT of the left clavicle (**A**) Osteosclerosis and bone expansion of the left clavicle extending to the sternoclavicular joint. (**B**) Osteitis and hyperostosis of the left sternoclavicular joint.

**Fig. 2:**
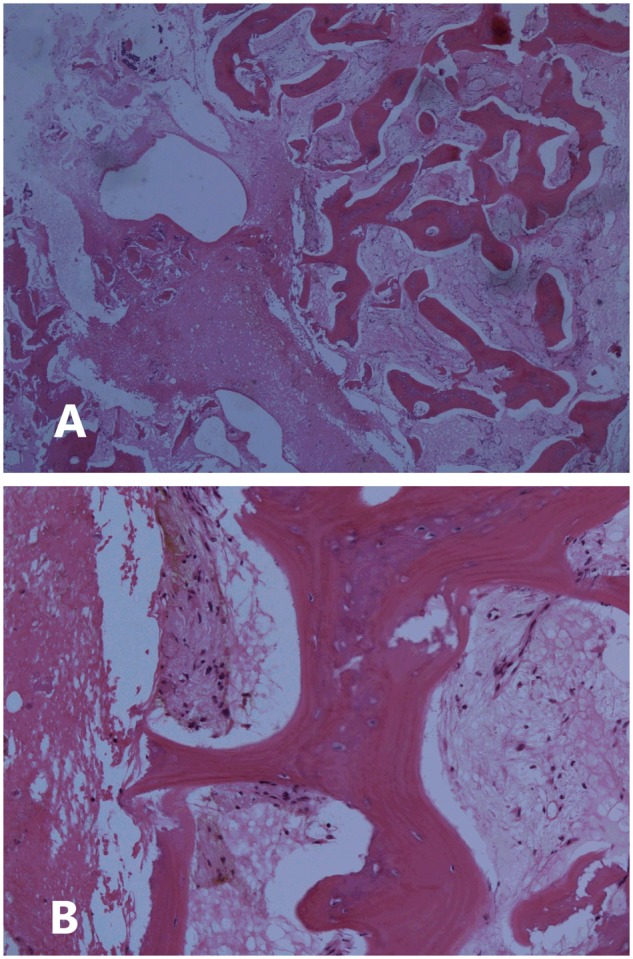
Bone biopsy (**A**) Low-power view, with several spiculated portions of lamellar bone with intertrabecular oedema and fibrin. (**B**) High-power view of the intertrabecular oedema, fibrin and mild chronic inflammation, including haemosiderin-laden macrophages.

Laboratory investigations at presentation were all within normal limits, including HLA-B27 negative. Testing was not performed for genetic autoinflammatory diseases. CT imaging compared with imaging from 5 years earlier demonstrated progression of a lytic lesion at the left medial clavicle to diffuse sclerosis and subchondral erosions extending across the sternoclavicular joint (Fig. 1). Bone scintigraphy initially showed only increased activity at the left sternoclavicular joint, which had progressed to the mid-left clavicle. An open incisional biopsy was performed on the left medial clavicle (Fig. 2). Samples sent for bacterial culture grew *S. saprophyticus* and *C. acnes*, both susceptible to penicillin. Mycobacterial and fungal cultures were negative.

Management was initially with NSAIDs, with some response. His chronic osteitis was treated with a prolonged course of penicillin V p.o. and penicillin G i.v. for a total of 2 years. The patient received one dose of bisphosphonate i.v. during the course of his antibiotic regimen. DMARDs were not pursued. On completion of antimicrobial therapy, he underwent a bone scan that demonstrated ongoing uptake by the left clavicle consistent with SAPHO, with no white blood cell accumulation on white blood cell nuclear medicine scan to suggest osteomyelitis. Since completion of antimicrobials and bisphosphonate therapy, his pain has dramatically improved, and he has not had any relapse on follow-up for the past 10 years.

Diagnosis of SAPHO syndrome is based on criteria described by Kahn & Kahn [2]. Common clinical manifestations include inflammation and excessive bony growth, particularly at the sternoclavicular joints. Synovitis generally affects large joints, and there is a common association with axial arthropathy. Dermatological pustular manifestations generally precede skeletal manifestations and may be either psoriatic (palmoplantar pustulosis) or acneiform (acne conglobate, acne fulminans or hidradenitis suppurativa) [3]. The disease course is generally chronic with intermittent flares, although it can be highly variable. Radiographic early lesions are osteolytic and become progressively sclerotic. Scintigraphy demonstrates increased uptake at bony lesions, with a pathognomonic bull’s head sign denoting increased uptake at the bilateral sternoclavicular joints and the sternum [4].

The aetiology of SAPHO is thought to be related to genetic, immunological and infectious factors. One theory for the pathogenesis of SAPHO is that it could be considered a seronegative spondyloarthropathy. An alternative theory is that low-virulence pathogens might cause infectious bone lesions or might trigger autoimmune inflammation of the bone [5]. *Cutibacterium* *acnes* is the most frequently identified pathogen on microbiological testing and was isolated in two-thirds of cases in one series [3]. Molecular studies have demonstrated that *C. acnes* has chemoattractant properties and can trigger IL (particularly IL-1) and TNF-α release to amplify the body’s immune response. Other isolated pathogens have included *Staphylococcus* *aureus*, *Haemophilus parainfluenzae*, *Actinomyces* and *Treponema pallidum* [5]; however, *S. saprophyticus* has not previously been identified in the literature as a contributing pathogen.

First-line treatment of SAPHO is usually with NSAIDs and/or analgesics for symptomatic control [6]. Antibiotics targeted at *C. acnes* or *S. aureus* have been tried, particularly doxycycline, trimethoprim–sulfamethoxazole, clindamycin and azithromycin; however, the effect is often not durable after the antibiotics have been stopped [5]. Bisphosphonates have shown some sustained benefits, related to their inhibition of bone turnover and possible anti-inflammatory effects [3]. DMARDs have been widely used, but have not shown significant efficacy for remission. In patients with chronic courses, biologic agents have been prescribed with increasing success to inhibit TNF-α and IL targets implicated in the immunological pathogenesis of SAPHO [6]. Our patient achieved remission with prolonged antibiotic and bisphosphonate therapy, which might support the proposed infectious theory in the pathogenesis of SAPHO syndrome.
